# Peritoneal Dialysis in Dengue Shock Syndrome May Be Detrimental

**DOI:** 10.1155/2012/917947

**Published:** 2012-03-15

**Authors:** Chulananda D. A. Goonasekera, Bhagya G. Thenuwara, Ranjith P. V. Kumarasiri

**Affiliations:** Department of Anesthesiology, Faculty of Medicine, University of Peradeniya, Peradeniya 20400, Sri Lanka

## Abstract

Dengue shock syndrome is the most severe form of Dengue that can be fatal. Nonresponders to standard therapy need intensive care. This paper outlines the clinical features, complications, and outcomes of Dengue Shock Syndrome not responding to standard therapies and needing supportive care in a tertiary referral intensive care unit of a developing country. Nearly one-third die within 3 days of admission to ICU. Peritoneal dialysis predicts the worst outcomes.

## 1. Introduction

Dengue is an important arthropod-borne viral disease of humans [1]. Its presentation varies from a mild viral syndrome to hemorrhagic fever and severe shock. Its severe forms (hemorrhagic fever and shock syndrome) may lead to multiorgan involvement and death. Dengue Shock Syndrome (DSS) is characterized by a massive increase in systemic capillary permeability with consequent hypovolemia [2]. The mortality rate in Dengue Shock Syndrome ranges from 6 to 30 percent, most commonly reported in children. Fluid resuscitation is critical with added support for failing organs [3].

In the recent past, Sri Lanka has experienced a surge of the disease reaching epidemic proportions associated with a probable change in the virus strain to a more virulent form [4, 5]. In this context we have also noted a rise in the number of cases with severe forms of the disease needing intensive care. The Peradeniya ICU is a tertiary referral centre and it attracts a large number of above patients with Dengue Shock Syndrome in the region who do not respond to standard therapy with intravenous fluids, antibiotics, and supportive care including inotropes.

## 2. Methods

We collected demographic data of all patients referred for intensive care between January 2009 and June 2010 (18 months) and analyzed their outcomes in relation to the complications of DSS that ensued during their ICU stay and the therapies given. The diagnosis of Dengue has been established upon clinical grounds (WHO guidelines 2009) and treated accordingly by the referring physicians before admission to the ICU. On admission to ICU, all have had fever, vomiting 78%, abdominal pain 21%, cough 5%, and body ache among 3% of cases. Eleven patients have had serological tests and Dengue IgM was found to be positive amongst 72% and IgG in 50% of the tested. They all have had intravenous fluid therapies and antibiotics. During the course of therapy in ICU, 43.6% of cases received fresh frozen plasma, 21.8% cryoprecipitate, 32.7% blood, and 56.4% platelet transfusions. Furthermore, 36.4% of cases were mechanically ventilated for multiple reasons such as severe respiratory distress (FiO_2_ > 60%), RR > 40/min, myocardial failure needing inotropes for persistent hypotension despite adequate filling, that is, CVP above 12 cm of H_2_O. The decision for peritoneal dialysis was based on low urine output (<0.5 mL/kg/hour) detected over a period of time in an ICU as a trend despite resuscitation with fluids combined with a situation of fluid overload, high CVP, persistent hypotension, or severe ARDS. Neither the plasma creatinine (rather a delayed indicator of AKI) nor blood urea (not reliable in the presence of liver impairment) was used as a determining factor in implementing dialysis. Persistent hypotension in the presence of high CVP was interpreted as indication of myocardial involvement (with or without relative bradycardia, ECG changes) and was supported with inotropes, usually a combination of dobutamine and nor-adrenaline and with mechanical ventilation if oxygenation was compromised.. Thus, our data is based on a group of severely ill patients diagnosed to be suffering from Dengue Shock Syndrome and continuing to deteriorate despite intravenous therapies administered in the wards. Liver failure regime, that is, oral/NG metronidazole and lactulose was introduced in patients who were found to have raised transaminases. It should be noted that the number of ICU beds available (10) in our hospital was approximately 1.5% of the total beds and this may have delayed the admission of some cases due to rationing.

## 3. Results

**Figure 1:**
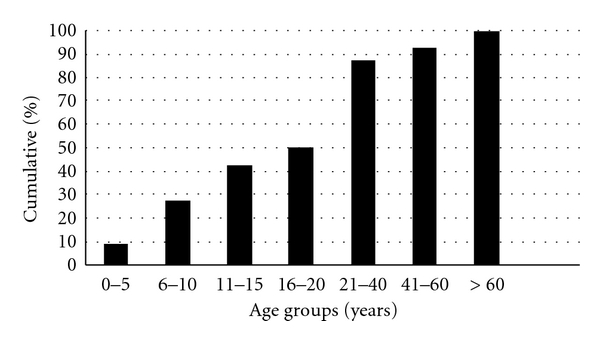
The cumulative age distribution of patients with Dengue shock syndrome needing intensive care.

Between January 2009 and June 2010, 54 cases (25 male) diagnosed of Dengue Shock Syndrome were admitted for treatment to the Peradeniya ICU, a tertiary referral center. Half of them were aged 20 or below as shown in Figure 1. On admission, all had received prior intravenous fluid therapies. Their mean (SD) PCV was 44.5% (5.5), WBC 7.7×10^9^/L (4.6), Platelet count 22 10^9^/L (17), respectively.

Of the 54, 16 died (mortality 29.6%). Most deaths (88%) occurred within 3 days of admission to the ICU (Table 1). The survivors needed intensive care for a median of 2 days (range 1–8) before being discharged to the referring wards for convalescence.

It is also noteworthy that 62.5% deaths occurred below the age 20 (see Table 2). Although we were unable to prove that risk of death was higher in children, a higher incidence of deaths (38% as opposed to 22%) was observed below the arbitrary cutoff age of 20 years.

To evaluate the risk of death according to the manifesting complications we performed a bivariate analysis and calculated the odds ratios (ORs) and confidence intervals (CI). OR and CI provide information on the strength (level of statistical significance) of association between the complications and the occurrence of deaths. Whereever the numbers of subjects were too small, the Fisher's exact test was used to calculate the *P* value (Table 3).

We found that the complications of Dengue, namely, hemorrhage, pleural effusion, myocarditis, liver failure, and renal failure were independently linked with a 7–11 times higher risk of death compared to those without (Table 3). However, the wide confidence intervals indicated the higher variability of this observation.

The effect of treatment modality on the outcome (death) was evaluated with chi-square test (see Table 4). Chi-square test is widely used to evaluate the association between these predictor and outcome variables.

The results revealed that the treatment modalities, namely, the use of inotropes, mechanical ventilation, peritoneal dialysis, and the use of blood products were significantly associated with higher occurrence of deaths among these patients (Table 4). However, the use of steroids had no association with death.

Thereafter a discriminant analysis was used to classify the cases according to the values of categorical dichotomous-dependent variables. This analysis assesses the relative importance of the independent variables in classifying the dependent variable.

The standardized canonical discriminant function coefficients identified mechanical ventilation and peritoneal dialysis as therapeutic modalities significantly associated with the deaths of dengue patients presenting with Dengue Shock Syndrome (Table 5).

A similar discriminant analysis was used to assess the relative importance of complications and outcome. Renal failure and hemorrhage were identified as complications significantly associated with deaths in Dengue Shock Syndrome (Table 6).

## 4. Discussion

We have evaluated the mortality risk factors amongst a cross-section of patients in Dengue Shock Syndrome not responding to standard therapies and as a consequence in a clinical scenario confounded by previous therapies before admission to ICU. Dengue Shock Syndrome is a dangerous complication of the dengue infection and is associated with high mortality. Almost one-third of our study group received blood transfusions to counter their bleeding manifestations. Thus, we were seeing the worst cases of the spectrum.

The pathogenesis of shock in dengue is complex. Increased vascular permeability, together with myocardial dysfunction and dehydration due to capillary leakage, contribute to the development of shock, with resultant multiorgan failure. The onset of shock can be dramatic, and its progression relentless. The diagnosis is largely clinical and is supported by serology and identification of viral material in the blood. No specific methods are available to predict outcome and progression. As observed by Singhi et al. [6] the choice of fluids, inotropes, and techniques of organ support and careful fluid management is the mainstay of management.

We have recorded a 30% mortality risk for this unique group of patients with Dengue Shock Syndrome who had received prior medical therapies and was admitted to Intensive Care with further deterioration. Unfortunately, the fact that 50% of the patients who succumbed did so within the first 24 hours of admission to the ICU indicates their moribund state upon referral to the ICU. It should be noted that the presenting clinical status of these patients to the ICU was confounded by a variety of treatment regimens that were applied before admission to the ICU. For example, in a typical patient admitted with respiratory distress and hypoxia, the clinical picture would easily be modified by overzealous hydration with colloids such as dextran or hetastarch prior to the ICU referral. In this study we have not been able to assess the influence of prior therapy on outcome due to poor medical records received at the admission to ICU. It is however the general impression of the authors, that the influence of prior therapies could be an important determinant of outcome, especially because some patients were noted to have had a cumulative dose of hetastarch exceeding 25 mL/kg suggesting overload contributing to respiratory distress more than the disease itself. This is a very important aspect that cannot be overlooked in future studies of this nature.

There is a general impression that fatal dengue is commoner in the younger population compared to middle or old age [7, 8]. Although we recorded 62.5% deaths amongst patients aged 20 or below, we have no statistical evidence to support the notion that mortality is higher amongst children. This is because our age distribution also indicated that 50% of the age cohort admitted for ICU care was above the age of 20. Peradeniya ICU is multidisciplinary and there are no age restrictions in its admission policy and hence we presume that our data represent the population with Dengue Shock Syndrome with no age bias.

In our study, from amongst the dead, 88% expired during first three days of ICU care and the highest death rate was reported on day 1 (50% of total deaths). A similar study during an epidemic of dengue hemorrhagic fever in easternmost Indonesia showed a case fatality rate of 1.2% from a 172 suspected cases. They too observed that, the survivors needed a range of 1–8 days of ICU care [9] similar to the durations we observed. Another study from Mumbai during a dengue epidemic reported a case fatality rate of 16.6% amongst pediatric patients suffering from Dengue Shock syndrome [10].

Bleeding has been identified as one of the dreaded manifestations of concern that complicates the outcome of dengue [11]. Although our canonical discriminant analysis indicated that hemorrhage and renal failure were the dreaded complications associated with death, from amongst its main therapeutic modalities only peritoneal dialysis (PD) was associated with death. This suggested that the use of blood products has effectively mitigated the effects of hemorrhage upon outcome. However, peritoneal dialysis did not show a similar effect suggesting that PD may not be the most appropriate modality of therapy in these moribund patients with multiorgan failure. It is also our clinical observation that PD cycles in Dengue patients produced a relatively large fluid retrieval without the use of additional measures such as dextrose in dialysate fluid. These large negative balances were corrected with the use of stored plasma intravenously. It is likely that these patients had ascites fluid that was also removed by each dialysis cycle and this may have simulated the main problem of dengue, the “capillary leak.” Thus, peritoneal dialysis may have aggravated the clinical effects of continuing “capillary leak” leading to worsened outcomes. We had no facility for Continuous Renal Replacement Therapy (CRRT).

We also found that mechanical ventilation was also associated with death. Only 21% of patients who received mechanical ventilation in the ICU recovered in this study (4 out of 19). Since we had no facility for ECMO, it is difficult to comprehend whether mechanical ventilation is the best supportive mode of therapy to maintain oxygenation in DSS.

There was a significant relationship between dengue, complications, and the modes of therapies and outcome. Hemorrhage, pleural effusion, myocarditis, renal failure, and liver failure were all important predictors of the worst outcomes. A study conducted in Thailand implied the importance of detection of abnormal high transaminase enzyme among the patients with dengue infection since the consequently developed hepatic encephalopathy could be expected [12]. In our study 9 out of 12 patients who were treated with liver failure regime expired (75%).

Dengue induced Acute Kidney Injury (AKI) comprising creatinine increase, proteinuria, glomerulonephritis, and haemolytic uremic syndrome has been reported [13, 14] and also dengue-haemorrhagic-fever-(DHF-) induced AKI even in the absence of shock, haemolysis, or rhabdomyolysis [15]. In our study 8 out of 8 (100%) patients who were suspected as having renal failure expired despite peritoneal dialysis. Similar to our observation, Kuo et al. in the year 2002 reported a Dengue outbreak in Taiwan and noted that patients with renal failure (RF) carry a high mortality rate, that is, the morality rate RF group versus non-RF group was 28.6% against 1.2%; *P* < 0.001 [16].

Acute reversible myocardial dysfunction is the commonest documented cardiac complication in dengue. The variable incidences of dengue myocarditis had been postulated to be due to variable immunopathogenesis secondary to variations in serotypes. Dengue myocarditis is generally reversible with favorable outcomes if diagnosed and treated early [17, 18]. In our study 15 out of 27 patients (55.55%) who were suspected of having dengue myocarditis were treated with inotropes but they died during their ICU stay.

It has been reported that corticosteroids were no more effective than the placebo or the no treatment protocol for reducing the number of deaths, the need for blood transfusion, or the number of serious complications [19] or in achieving a higher rise of the platelet count in dengue infection [20]. We have observed the same. No specific therapy has been shown to be effective in improving survival [21].

## 5. Conclusion

Our study indicates that during this dengue outbreak, patients in DSS who were not responding to standard therapies and admitted ICU had a 30% risk of death. Peritoneal dialysis increases this risk to 100%.

## Figures and Tables

**Table 1:** Was the death early or late in ICU?

Day	No. of deaths	%
1	08	50%
2	03	19%
3	03	19%
5	01	06%
15	01	06%

Total	16	100%

**Table 2:** Distribution of ICU deaths by age categories (*1 missing data).

	Live discharges	Deaths	Total
≤20 years	16	10	26
>20 years	21	06	27

Total	37	16	53*

*χ* ^2^ = 1.66; df = 1; *P* = 0.198 (not significant).

**Table 3:** Complications ensued and outcomes amongst patients with Dengue shock syndrome in ICU (*Fisher's exact test).

Complications		Deaths	Discharges	Total	OR	CI	*P*
Hemorrhage	Yes	13	14	27	7.4	1.8–30.7	0.003
	No	3	24	27			
	Total	16	38	54			

Pleural effusion	Yes	13	14	27	7.4	1.8–30.7	0.003
	No	3	24	27			
	Total	16	38	54			

Myocarditis	Yes	8	6	14	5.3	1.4–19.8	0.02*
	No	8	32	40			
	Total	16	38	54			

	Yes	10	0	10			
Renal failure	No	06	38	44			<0.001*
	Total	16	38	54			

Liver failure	Yes	08	03	11	11.7	2.2–54.1	0.001*
	No	08	35	43			
	Total	16	38	54			

**Table 4:** Therapeutic modalities applied and outcomes of patients with Dengue Shock Syndrome receiving intensive care.

Therapies		Deaths	Discharges	Total	*χ* ^2^	.df	*P*
Inotropes	Yes	15	12	27	17.41	1	<0.001
	No	01	26	27			
	Total	16	38	54			

Mechanical ventilation	Yes	15	4	19	34.1	1	<0.001
	No	1	34	35			
	Total	16	38	54			

Liver failure regime	Yes	9	3	12	15.23	1	<0.001
	No	7	35	42			
	Total	16	38	54			

Peritoneal dialysis	Yes	8	0	8	22.3	1	<0.001
	No	8	38	46			
	Total	16	38	54			

Blood products	1	14	11	25	15.2	1	< 0.001
	2	2	27	29			
	Total	16	38	54			

Steroids	Yes	7	13	20	0.353	1	0.553
	No	9	24	33			
	Total	16	37	53			

**Table 5:** Standardized Canonical Discriminant functions between therapeutic modalities and outcome (*Wilk's lambda = 0.315; df = 2; *P* < 0.001).

Variables	Canonical discriminant function coefficients
Mechanical ventilation	0.885*
Peritoneal dialysis	0.566*
Liver failure regime	0.474
Blood products	0.233
Steroids	0.215
Inotropes	0.192

**Table 6:** Standardized Canonical Discriminant functions between complications and outcome.

Variables	Canonical discriminant function coefficients
Renal failure	.919*
Liver failure	.476
Pleural effusion	.417
Hemorrhage	.377*
Myocarditis	.368

* Wilk's lambda = 0.419; df = 02; *P* < 0.001.
